# Vedolizumab combined with surgical resection successfully treated perforating Crohn’s disease with peritoneal space to rectal and vaginal fistulas: a case report

**DOI:** 10.1186/s12876-021-01875-6

**Published:** 2021-07-29

**Authors:** Meng-Wu Chung, Chien-Ming Chen, Jun-Te Hsu, Ren-Chin Wu, Cheng-Tang Chiu, Chia-Jung Kuo, Ming-Yao Su, Puo-Hsien Le

**Affiliations:** 1 Department of Medical Education, Chang Gung Memorial Hospital, Linkou Branch, Taoyuan, Taiwan; 2 Department of Medical Imaging and Interventions, Chang Gung Memorial Hospital, Linkou Branch, Taoyuan, Taiwan; 3 Department of General Surgery, Chang Gung Memorial Hospital, Linkou Branch, Taoyuan, Taiwan; 4 Department of Pathology, Chang Gung Memorial Hospital, Linkou Branch, Taoyuan, Taiwan; 5 Department of Gastroenterology and Hepatology, Chang Gung Memorial Hospital, Linkou Branch, Chang Gung University College of Medicine, 5, Fu-Hsin Street, Guei-Shan District, Taoyuan City, 33305 Taiwan; 6 Taiwan Association of the Study of Small Intestinal Disease, Taoyuan, Taiwan; 7 Department of Gastroenterology and Hepatology, New Taipei City Municipal Tucheng Hospital, New Taipei City, Taiwan; 8 Liver Research Center, Chang Gung Memorial Hospital, Linkou Branch, Taoyuan, Taiwan

**Keywords:** Vedolizumab, Surgery, Crohn’s disease, Perforation, Fistula

## Abstract

**Background:**

Intestinal perforations and fistulas are common complications of Crohn’s disease. However, chronic perforation with peritoneal space to rectal and vaginal fistulas have not been previously reported.

**Case presentation:**

A 38-year-old female suffered from progressive lower abdominal pain, diarrhea and weight loss. Terminal ileal chronic perforation with intra-abdominal abscess, peritoneal space to rectal and vaginal fistulas were noted. The patient received surgical resection of the cecum and terminal ileum, and then vedolizumab treatment. Three months later, she had complete fistula closure, and her body mass index had increased from 13 to 22.

**Conclusion:**

Vedolizumab combined with stool diversion is effective at treating Crohn’s disease with chronic perforation and complex peritoneal space to rectal and vaginal fistulas.

## Background

Crohn's disease (CD) is characterized by transluminal inflammation of the bowel; penetrating complications are often observed, such as intra-abdominal abscesses or fistulas, which are present in approximately 10–30% of patients at the time of diagnosis [1–4]. However, to the best of our knowledge, no cases of peritoneal space to rectal and vaginal fistulas have been previously reported. The management of intra-abdominal abscess with complex fistulas is challenging and requires a multidisciplinary approach, including percutaneous abscess drainage, surgical resection and biological treatment [5]. The non-perianal fistula closure rate of tumor necrosis factor (TNF) antagonist is 13–28%, but limited data is available for vedolizumab [6, 7]. We present a patient who had CD with chronic terminal ileal perforation and peritoneal space to rectal and vaginal fistulas. The patient had complete fistula closure after a combination of surgery and vedolizumab treatment.

## Case presentation

This 38-year-old female patient, with a body mass index (BMI) of 13, had suffered from progressive lower abdominal pain and intermittent fever for 3 months. She also had lost 32 kg of her body weight within 5 years. This time, the patient complained of progressive watery diarrhea for 2 weeks, and then visited our emergency department in November 2018. Due to her history of hyperthyroidism, she was first treated for a thyroid storm, and then admitted for further investigation due to her normal thyroid function.

**Fig. 1:**
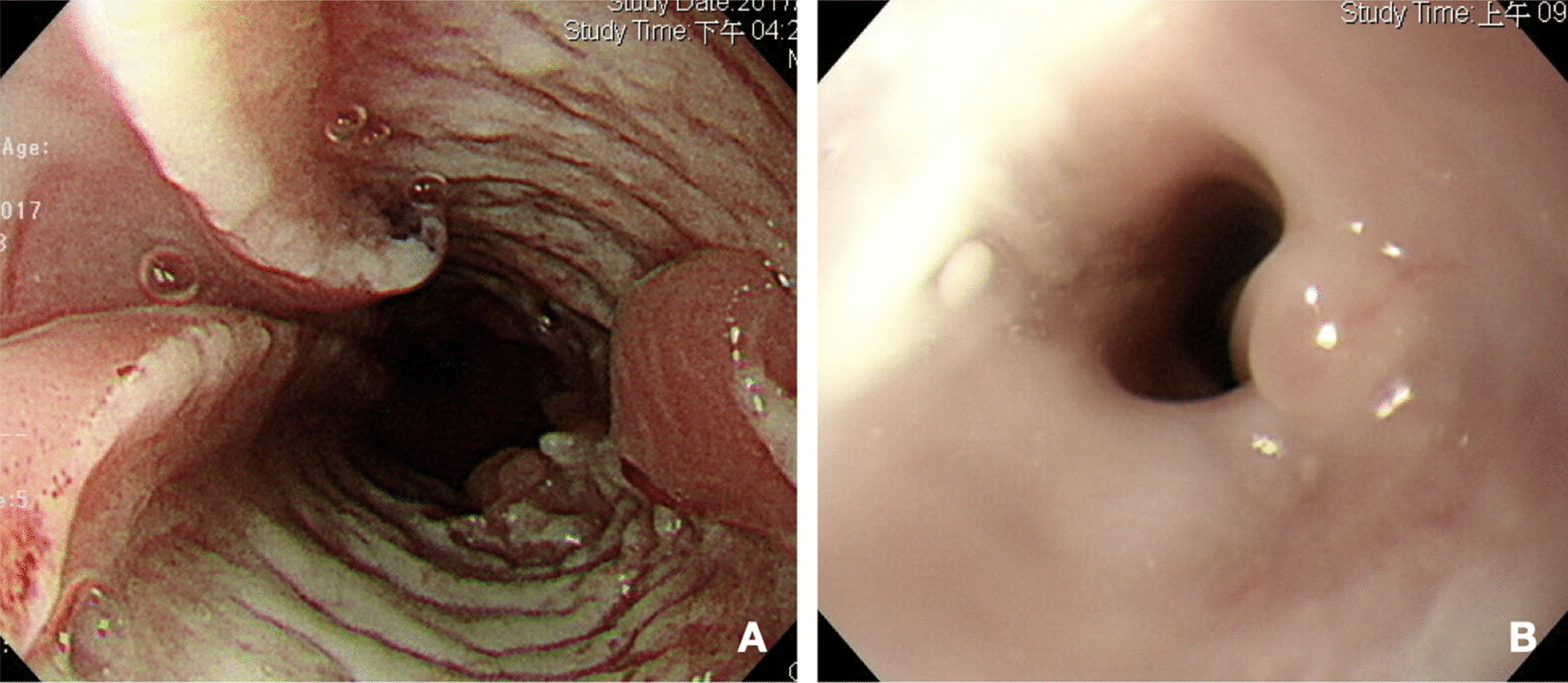
Esophagogastroduodenoscopy (EGD). **A** Esophageal deep ulcer, 33–38 cm from the incisor. **B** Esophageal stricture after 3 months of proton pump inhibitor treatment

The patient’s history of past illness includes an episode of esophageal ulcer, 33–38 cm level from the incisor, diagnosed in December 2017, status post proton pump inhibitor treatment, complicated with stricture in March 2018, status post endoscopic dilatation in August 2018 (Fig. 1). Pathologist found granulation tissue with acute and chronic inflammation with necrotic tissue. Besides, she also had hyperthyroidism under methimazole use since 2002 and history of coronary arterial disease, one vessel disease, right coronary artery, status post two bare metal stents and one drug-eluting stent insertion in January 2016 and February 2017, under aspirin use.

**Fig. 2:**
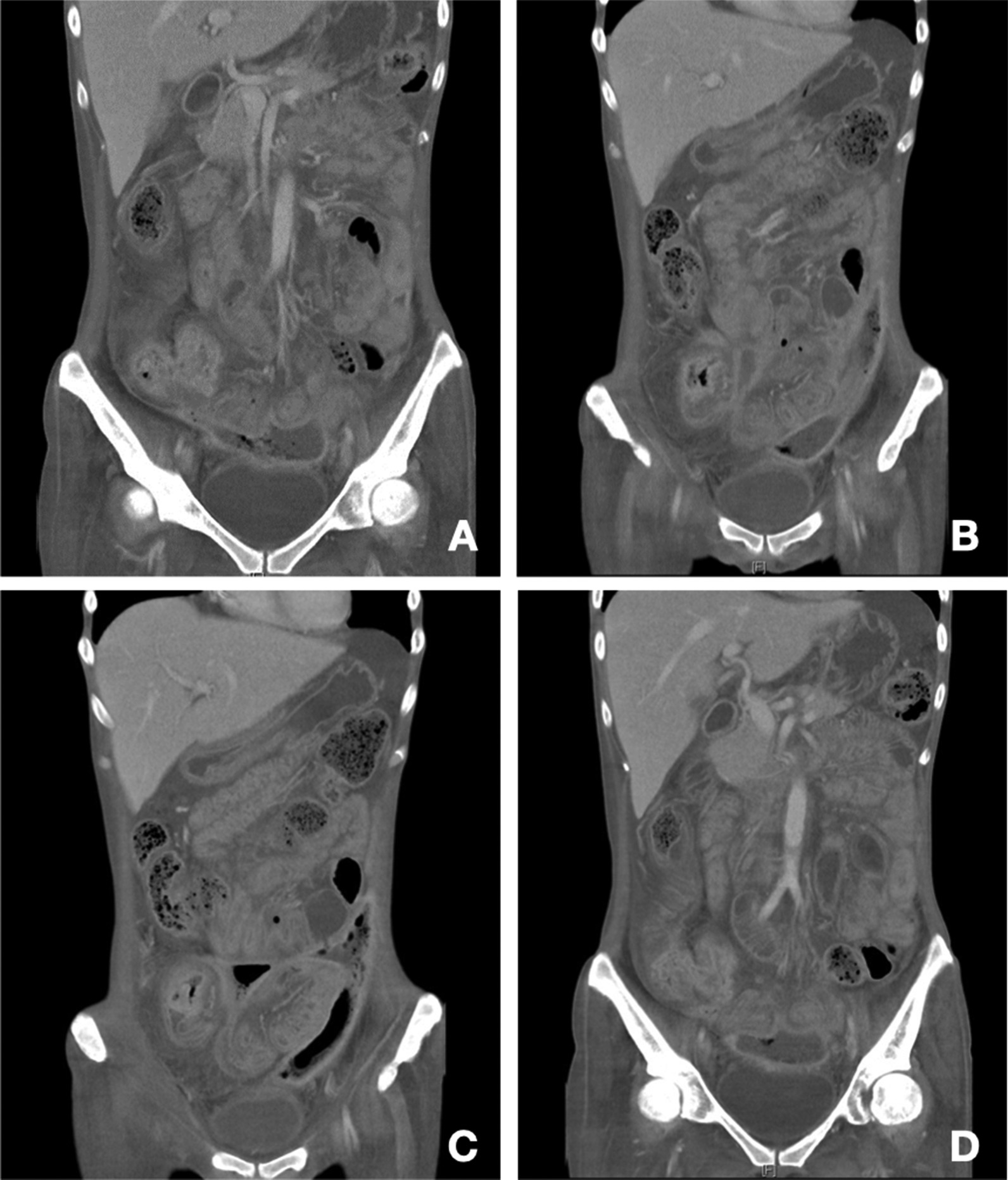
Computed tomography of the abdomen at diagnosis. **A** Wall thickening over the ascending colon, cecum and terminal ileum. **B** Suspicious terminal ileal perforation tract with a thick enhanced wall. **C**, **D** Irregular shaped fluid and gas collection with a thick, enhanced wall, extending from the upper to lower peritoneal cavity and cul-de-sac

**Fig. 3:**
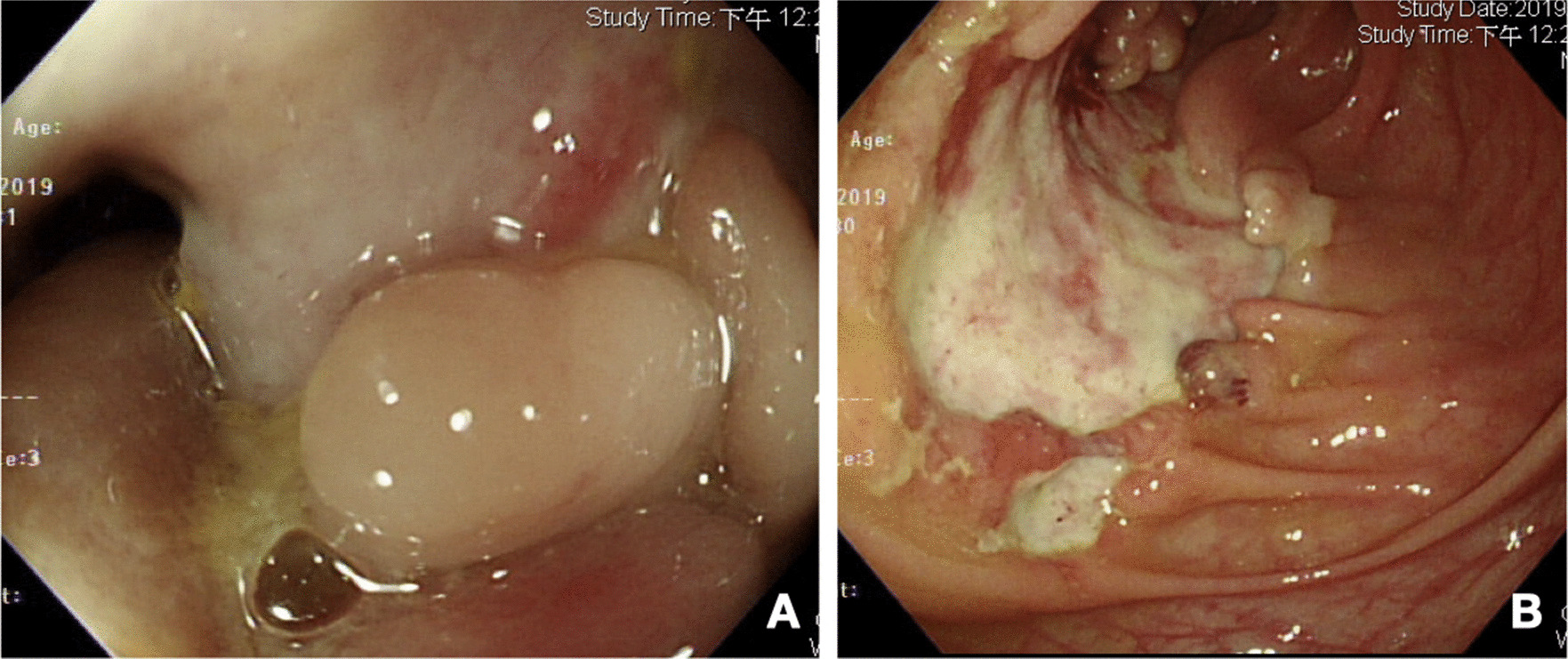
Colonoscopy at diagnosis. **A** Ileocecal valve ulcer with stricture. **B** Scending colon ulcer

**Fig. 4:**
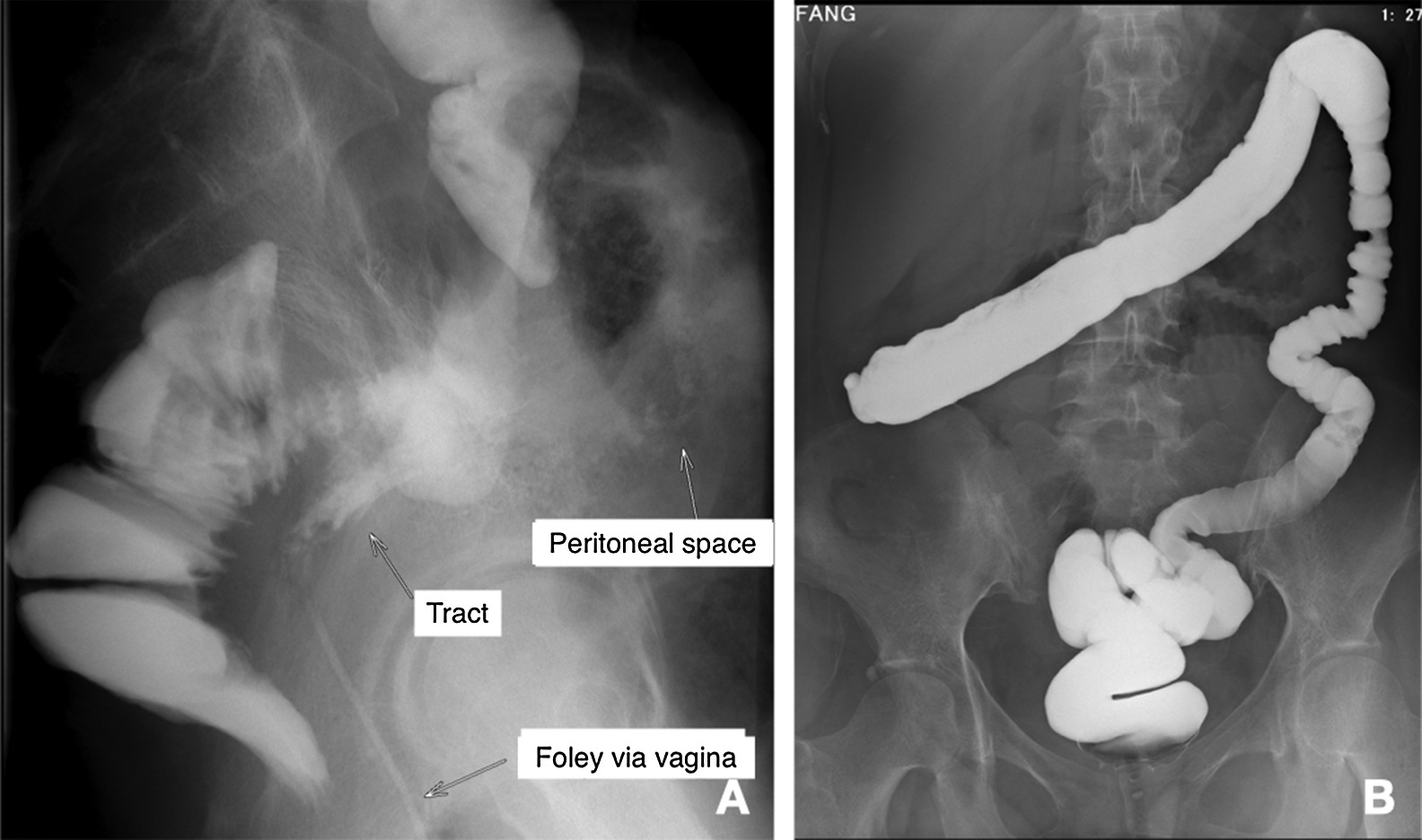
Lower gastrointestinal (LGI) study. **A** The vagina was marked by a Foley catheter. It showed peritoneal space to rectal and vaginal fistulas. **B** No more fistula tract was noted after 6 months treatment

After admission, physical examination found lower abdominal tenderness and tympanic sound to percussion, but no peritoneal signs. Laboratory survey revealed white blood cell count of 17,600/μL, segment 86%, myelocyte 0.5%, band 1%, lymphocyte 7%, monocyte 5.5%, hemoglobin 5.1 g/dL, platelet count 79,5000/μL, alanine aminotransferase (ALT) 9 U/L, sodium (Na) 127 mEq/L, potassium (K) 3.2 mEq/L, C-Reactive protein (CRP) 122.3 mg/L, free-T4, 0.91 ng/dL and thyroid-stimulating hormone (TSH)1.159 uIU/mL. Tests for cytomegalovirus (CMV) DNA, Epstein-Barr virus (EBV)- viral capsid antigen (VCA) IgM, human immunodeficiency virus (HIV) antibodies (Ab), amebic Ab, and cultures for Salmonella, Shigella, Campylobacter and pus cells in the stool were all negative. Positive CMV IgM, CMV IgG, EBV-VCA IgG and occult blood were noted. Computed tomography (CT) revealed wall thickening over the ascending colon, cecum and terminal ileum (Fig. 2A), a suspicious terminal ileal perforation tract with a thick enhanced wall (Fig. 2B) and irregular shaped fluid and gas collection with a thick, enhanced wall, extending from the upper to the lower peritoneal cavity and cul-de-sac, which measured up to 14 cm (Fig. 2C, D). Antibiotic treatment with piperacillin/ tazobactam was then administered and CT guided drainage was arranged. Ganciclovir IVF was also prescribed from 2nd December 2018 to 17th January 2019 due to the CMV IgM positive, DNA negative finding. Abscess culture grew *Enterococcus faecium* and *Candida glabrata*. Antifungal treatment with anidulafungin was prescribed from the 7th December 2018 to 24th January 2019. A colonoscopy showed deep ulcers over the ileocecal valve (ICV) (Fig. 3A) and proximal A-colon with ICV stricture (Fig. 3B). The pathologist noted ulcers, and the remaining mucosa showed crypt branching, crypt shortening, and pyloric metaplasia. This was compatible with Crohn’s disease with a negative immunohistochemistry for CMV, which was performed with a monoclonal antibody directed against the CMV pp65 antigen (Novocastra™ lyophilized mouse monoclonal antibody; Leica Microsystems, Wetzlar, Germany). This led to a diagnosis of Crohn’s disease with a Montreal classification of A2L3L4B2B3, a Crohn’s disease activity index (CDAI) of 623 points and a Harvey–Bradshaw Index (HBI) of 22 points. After infection control, oral prednisolone 20 mg/D and azathioprine 25 mg/D were prescribed. We stopped the azathioprine treatment due to nausea and vomiting side effects. The patient was discharged with oral prednisolone 20 mg and oral levofloxacin in January 2019. One month later, she complained of stool-like discharge from the vagina. A fistulogram through the vagina revealed peritoneal space to rectal and vaginal fistulas (Fig. 4A).

The final diagnosis was Crohn’s disease, with a Montreal classification of A2L3L4B2B3, which was complicated by an esophageal ulcer with stricture and chronic terminal ileal perforation with peritoneal space to rectal and vaginal fistulas.

**Fig. 5:**
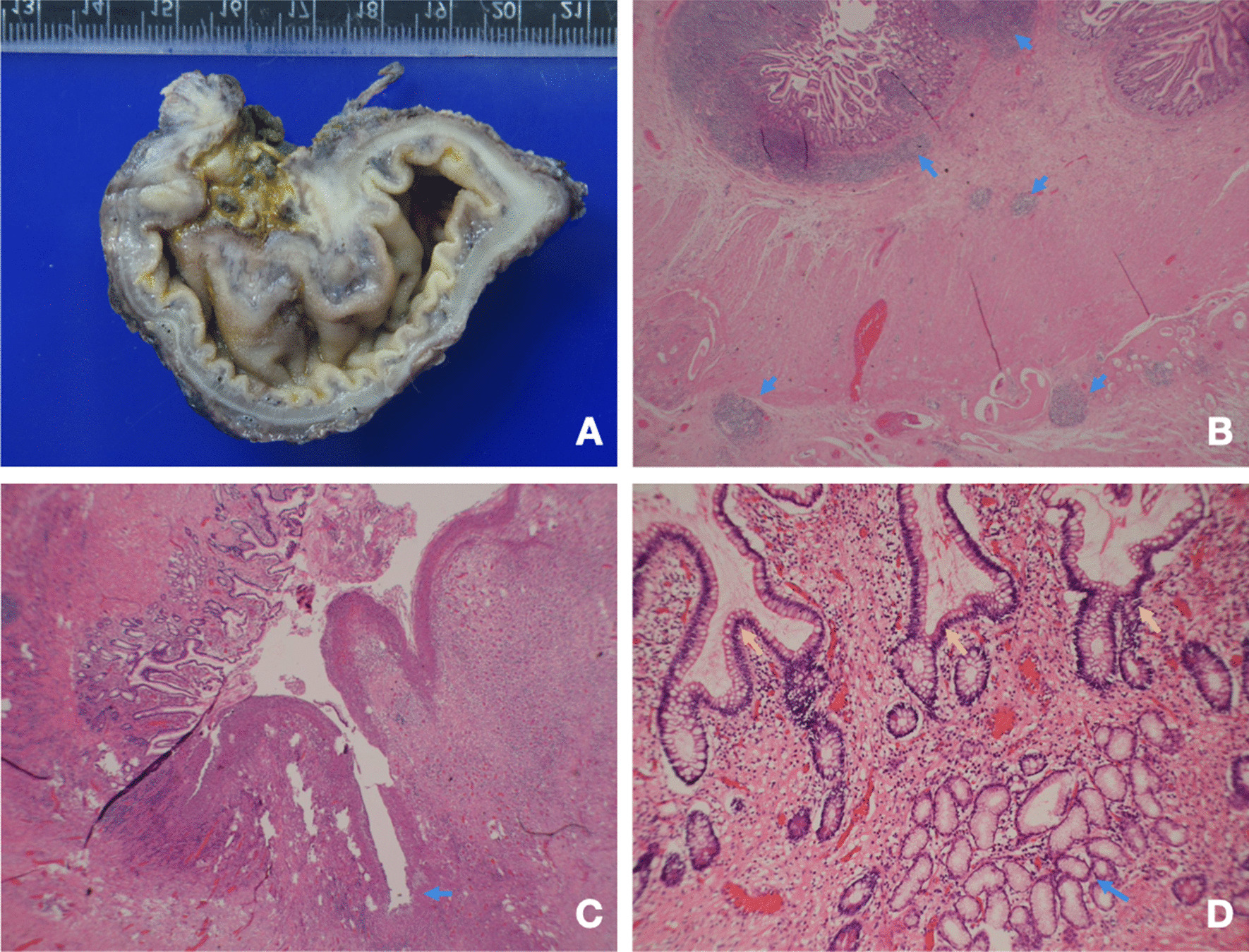
Pathological presentation of Crohn’s disease. **A** Dense fibrous fistula from the terminal ileum. **B** Arrow: lymphoid follicles, demonstrating transmural involvement. **C** Arrow: a fissure. The remaining mucosa on the left shows marked crypt distortion. **D** Blue  arrow: crypt branching. Yellow arrow: pyloric metaplasia (both are features of chronicity)

The patient received surgical resection of the cecum, terminal ileum and chronic perforated tract over the terminal ileum, and ileo-colostomy side to side anastomosis and an end-ileostomy on the 1st April 2019. The pathologist noted that the entire specimen measured 9.5 × 8.0 × 5.5 cm. The intestine showed ulceration, pseudopolyps, and a fistular tract surrounded by purulent exudate (Fig. 5A). These microscopic findings were compatible with the diagnosis of Crohn’s disease (Fig. 5B–D). She had no more stool like material passing out from her vagina 1 week later. The patient was prescribed vedolizumab treatment with standard induction and maintenance dose (300 mg in 0th week, 2nd week, 6th week, and then every 8 weeks) on the 24th April 2019.

In July 2019, the patient underwent colonoscopy examination, which only showed mild inflammatory activity over the blind end, and a lower gastrointestinal (LGI) series also noted no more fistula. Closure of the end-ileostomy with an end-to-side ileo-ascending-colon anastomosis was performed in August 2019. A lower gastrointestinal tract X-ray examination was arranged, which revealed no remaining fistula or obstruction (Fig. 4B). The patient regained 23 kg of body weight, with her BMI increasing from 13 to 22. Her CDAI dropped from 623 to 4 points and her HBI fell from 22 to 0 points.

## Discussion and conclusion

The management of nonperianal penetrating Crohn’s disease with abscess is always challenging because not only the inflammation but also the infection [8] needs to be controlled. Percutaneous drainage with antibiotic treatment can prevent 30% of Crohn’s disease patients with intra-abdominal abscesses from requiring surgery [9]. Anti-TNF alpha agents, especially infliximab, with the strongest evidence in treating fistulizing Crohn’s disease, with a 14–25% response rate for internal fistulas [10–13], and a 30–50% fistula closure rate for kinds of fistulas [14–16]. Vedolizumab, a gut-selective α4β7 integrin antagonist, also showed a 31% fistula closure rate at Week 52 in the GEMINI 2 trial [16], although there was a small sample size and limited other evidence. However, compared with anti-TNF antagonists, vedolizumab had less severe infection and adverse effects in a real world study [17]. Therefore, in patients with Crohn’s disease who have an infection or are at high risk of progressive infection, it might be the optimal choice. In this complex case, early percutaneous drainage, surgical diversion and early biological treatment led to a favorable outcome.

In conclusion, a multidisciplinary approach is critical when treating perforating Crohn’s disease with intra-abdominal abscess and complex fistula, to ensure successful management of the condition. As far as biologics is concerned, vedolizumab could be the optimal choice due to the lower risk of worsening the infection and its fair ability to control the inflammation. Larger well-designed studies are required to thoroughly assess the effectiveness of vedolizumab in treating complex fistulizing Crohn’s disease.

## Data Availability

The datasets used and/or analysed during the current study are available from the corresponding author on reasonable request.

## Abbreviations

CD: Crohn’s disease
TNF: Tumor necrosis factor
BMI: Body mass index
ALT: Alanine aminotransferase
Na: Sodium
K: Potassium
CRP: C-reactive protein
TSH: Thyroid-stimulating hormone
CMV: Cytomegalovirus
EBV: Epstein–Barr virus
VCA: Viral capsid antigen
HIV: Human immunodeficiency virus
Ab: Antibody
CT: Computed tomography
IVF: Intravenous fluid
ICV: Ileocecal valve
CDAI: Crohn’s disease activity index
HBI: Harvey–Bradshaw index
LGI: Lower gastrointestinal
